# Sensory profiling and consumer acceptability of new dark cocoa bars containing Tuscan autochthonous food products

**DOI:** 10.1002/fsn3.523

**Published:** 2017-11-28

**Authors:** Claudio Cantini, Patrizia Salusti, Marco Romi, Alessandra Francini, Luca Sebastiani

**Affiliations:** ^1^ Trees and Timber Institute ‐ National Research Council of Italy CNR‐IVALSA Follonica Italy; ^2^ Dipartimento di Scienze della Vita Università degli Studi di Siena Siena Italy; ^3^ Institute of Life Sciences Scuola Superiore Sant'Anna Pisa Italy

**Keywords:** autochthonous, consumer test, organoleptic, panel test, sensory profiling

## Abstract

A new set of cocoa bars named Toscolata^®^ were developed containing top‐quality extra virgin olive oil, dried apples cultivars, and chestnut flour. The present work has been conducted to define the sensory profile of these products through tasting by trained experts and consumers to study the acceptability, preference, and quality perception. The four sensorial profiles of the bars differed in the level of persistence, bitterness, aromaticity, acidity, astringency, and tastiness. In particular, the sour attribute could be traced to the presence of dried apple. Bars containing apple and chestnut flour obtained higher acceptance ratings, compared to those with extra virgin olive oil. The bar with chestnut flour was preferred by consumers who considered it to be sweeter due to the presence of natural sugars, which lowered the bitter sensation of cocoa. These results showed that the selection of the preferred bar by consumers was mainly based on the level of bitterness and, in particular, elderly consumers expressed a strong preference for the sweetest product. As far as we know, this is the first study comparing the results of a panel of expert tasters with that of consumers in the tasting of dark chocolate.

## 1 INTRODUCTION

In recent years, consumers have become increasingly aware of foods of high in nutritional value with organoleptic qualities related to traditional production.

Tuscany is the regional base of several typical agricultural products; many of them are labeled under Protected Designations of Origin (PDO) or Protected Geographical Indications (PGI). Other traditional products are not so easy to be marketed because they do not meet market standards. For example, some fruits do not reach the desiderate size, color, and firmness or may have a very short season. Thus, it would be useful to find new strategies for their commercialization selecting new formulations, novel methods of transformation by which increase the economic value and the income of the producers.

One confectionary products preferred by consumers is cocoa and its derivatives. They are consumed for pleasure, acting as a stimulant, relaxant, or potentially an antidepressant (Parker, Parker, & Brotchie, 2006). Cocoa is appreciated not only for hedonistic properties (Beckett, 2000), but also for health benefits due to its high content of antioxidants. Many recent studies showed a correlation between consumption of dark chocolate and the reduction in cardiovascular risks with positive action against hypertension, free radicals, and low‐density lipoproteins oxidation (Ariefdjohan & Savaiano, 2005; Arranz et al., 2013; Ding, Hutfless, Ding, & Girotra, 2006; Ellam & Williamson, 2013; Engler & Engler, 2006; Ried, Sullivan, Fakler, Frank, & Stocks, 2010).

Cocoa can be combined with fruits, vegetable oil, and many other ingredients. These combinations affect the texture and consequently consumer acceptability (Beckett, 1994) which is also influenced by variations in the proportions of ingredients or processing (Jackson, 1999). As reported by Rozin and Fallon (1987) “food acceptance are motivated by a combination of sensory‐affective reasons ideational notions and safety concerns”. Consumer opinion represents an effective quality‐level assessment; however, for profiling a new product it is necessary to have the judgment of a trained panel which assures accuracy, sensibility, and repeatability.

Although the consumption of high‐quality cocoa products and chocolates are increasing, the literature on sensory properties of dark chocolate is scarce and there is a lack of any official organoleptic evaluation procedure. Several studies have addressed consumer perceptions and sensory properties of milk chocolate (Chapman, Rosenberry, Bandler, & Boor, 1998; Hough & Sanchez, 1998; Thompson, Drake, Lopetcharat, & Yates, 2004; Thompson, Gerard, & Drake, 2007; Yanes, Duran, & Costell, 2002) and diabetic/reduced calorie chocolate (de Melo, Bolini, & Efraim, 2009). Some studies have also been conducted to understand the acceptance of dark chocolate in Belgium and Poland (Januszewska & Viaene, 2001), or filled chocolate in Brazil (Miquelim, Behrens, & Lannes, 2008). Recent research addressed the sensory profile of Italian cocoa products obtained using typical ingredients and techniques (Lanza, Mazzaglia, & Pagliarini, 2011; Speziale, Vazquez‐Araujo, Mincione, & Carbonell‐Barrachina, 2010), but consumer preference was not explored.

In the 2013, the Tuscany region financed applicative research aimed at improving the agricultural economy and increasing the revenue of typical local foods. In collaboration with a private company, we developed a new set of cocoa bars containing top‐quality extra virgin olive oil, dried apples produced by old autochthonous cultivars and chestnut flour. The prototypes of these new cocoa products named Toscolata^®^ have been subjected to several studies with different approaches. The present work has been performed to define the sensory profile made by trained experts of these new products and to study the consumer acceptability, preference, and quality perception.

## 2 MATERIALS AND METHODS

### 2.1 Samples

Seven cocoa bars prototypes were used for this study: five containing dried apples of different cultivars. Three of them “Mora,” “Nesta,” and “Ruggine” are traditional varieties from Tuscany while “Stayman” and “Golden Delicious” are internationally cultivated and were used as a comparison. Another bar was made using top‐quality extra virgin olive oil, and the last bar by adding PGI Monte Amiata chestnut flour.

Each ingredient was obtained from local organic producers and selected for their high antioxidants levels (oil and apples) or organoleptic features (chestnut flour).

The samples were manufactured in the Vestri chocolate laboratory located in Arezzo (http://www.vestri.it) using the best mixture of cocoa beans produced directly by Vestri in their organic farm in Santo Domingo. All the production conditions (mixing, refining, conching, tempering, molding, and cooling) were set to maintain the highest organoleptic features of the original cocoa beans and ingredients. The composition of the bars is given in Table 1, where the abbreviations for each bar used in the text are also provided. Bars were packed in 40 g size and stored at 4°C.

**Table 1:** Sample coding, cocoa content, and ingredients of the cocoa bars

Sample code	Cocoa content	Ingredients	
DAG	70%	Cocoa mass, cane sugar, vanillin, cocoa butter, dried Golden Delicious apple	
DAM	70%	Cocoa mass, cane sugar, vanillin, cocoa butter, dried Mora apple	
DAN	70%	Cocoa mass, cane sugar, vanillin, cocoa butter, dried Nesta apple	
DAR	70%	Cocoa mass, cane sugar, vanillin, cocoa butter, dried Ruggina apple	
DAS	70%	Cocoa mass, cane sugar, vanillin, cocoa butter, dried Stayman apple	
CHF	70%	Cocoa mass, cane sugar, vanillin, cocoa butter, PGI Monte Amiata chestnut flour	
EVO	70%	Cocoa mass, cane sugar, vanillin, cocoa butter, PGI Tuscan extra virgin olive oil	

### 2.2 Experimental design

Several rapid techniques for the determination of consumer preference and food quality perception have been recently introduced (Varela & Ares, 2012) to speed up the process of sensory characterization and product profiling. Since our products had a similar composition (a base of 70% cocoa for all), we preferred to use a time‐consuming classic three‐step approach that included (1) a tasting and discussion session with sensory professionals following the method ISO 11035:1994, (2) a pilot sensory evaluation by panel composed of experts, and (3) a large‐scale consumer test focusing on acceptability and preference of cocoa bars.

### 2.3 Pretest

The aim of this stage was the identification of the descriptors to be used for the evaluation of each aspect of the products. A list of descriptors taken from the literature (Lanza et al., 2011; Sune, Lacroix, & Huon De Kermadec, 2002; Thamke, Durrschmid, & Rohm, 2009) was provided to the members of the round table. Four preparatory sessions were conducted with selected standards and the assessors were asked to evaluate individually if the terms were suitable or whether it was necessary to introduce new terms. Following this step, a discussion guided by the panel leader was conducted taking into consideration all the responses of the assessors and sensory features of the innovative products. These sessions were conducted to reduce the list of the descriptive terms and to define the organoleptic evaluation sheet to be presented to the professional panel following the method reported in the paragraph 6 of ISO International Organization for Standardization (1994) ISO 11035:1994 (E). 11035:1994 (E).

### 2.4 Panel test

Before the test, an introduction was delivered to the panelists to explain the objective of the research, the precise meaning of each cocoa attributes and to give examples of the grade of intensity. Several cocoa products were used as standards (dried cocoa beans; 100% dark chocolate; milk chocolate). All of the samples to be assessed were taken out of the refrigerator 24 hr before each session. Each cocoa bar was cut into 6.5 g servings, placed on to plastic plates codified with three‐digit random numbers and served at room temperature (22°C) to the panelists without specifying the formulation. As recommended, water was used for cleansing the palate between samples.

The sensory evaluation was conducted in two replicate sessions by a panel of the Grosseto Chamber of Commerce composed of 10 experienced judges. In the first session, the products containing “Mora,” “Nesta,” “Ruggine,” “Stayman,” and “Golden Delicious” (DAM, DAN, DAR, DAS, and DAG, respectively) were evaluated to understand if there was any difference between local and international dried apples added to the cocoa. In the second session two apple bars were evaluated together with extra virgin olive oil (EVO) and chestnut flour (CHF). Attributes were expressed on a 9‐cm line scale and quantified by measuring the distance of the mark from the origin (Dever, Macdonald, Cliff, & Lane, 1996).

### 2.5 Consumer test

After the panel test, only four bars were submitted to the consumer test. The aim of this step was to determine the acceptability and level of preference of the new cocoa bars by a representative sample of the consumer population. The test was conducted for 3 days in different locations: Pisa, Siena, and Follonica. The consumers were recruited during the “European Researchers’ Night” (http://ec.europa.eu/research/researchersnight/index_en.htm) where young, adults, and families interact directly with researchers.

The consumers, both male and female, were selected by two main criteria: 18–80 years old and are frequent consumers of cocoa products. A total of 182 people took part in the study on voluntary basis. They were first asked to complete a questionnaire regarding their general sociodemographic information (see Table 5). Each sample consisted of 3.2 g of the cocoa bar, placed in transparent plastic bowl at room temperature, codified with a three‐digit code and served in a random order. Consumers were asked to taste cocoa bars and to express an acceptance score on a 1–9 hedonic scale from 1: “I completely dislike” to 9: “I like very much”. Afterwards, the consumers were asked to focus on the sample they preferred the most and to select which attributes contributed to their choice. They could choose one or more sensorial descriptors among those considered to be discriminant after statistical analysis of the data provided by the professional panel and suggested during the interview.

### 2.6 Statistical analysis

Geometric means were calculated using the level of intensity and frequencies of each descriptor during the pretest for selecting the attributes to be subsequently used in the panel test. The median of each sensory attribute was calculated for each of the panelist and generalized procrustes analysis (GPA) was applied to analyze the dataset to standardize the scale of evaluation. Principal component analysis (PCA) was applied to the dataset from the panel test to select the most discriminant attributes and to study differences among all of the samples. Principal component analysis was also applied to discriminate groups of consumers on the base of sociodemographic differences. A Pearson's chi‐square test was used to study the differences in the distribution of cocoa bar preference within the groups of consumers. All the statistics were performed by Systat 11 statistical program (Systat Software Inc. Richmond, CA, USA).

## 3 RESULTS

### 3.1 Pretest Results

A total of 38 potential sensory terms were used for the preliminary description of cocoa prototypes (Table 2). During the round table sessions among the assessors another three attributes were introduced: two of them, “tastiness” and “aromaticity”, were chosen to define the overall intensity of the respective taste and aroma without any precise qualitative definition. The third term “vegetal” was defined as the smell of green fruit. After tasting and the following discussion on cocoa bars, 13 terms listed in Table 2 were eliminated because they correlated with specific ingredients not used in our recipes (butter, cinnamon, vanilla, nutty, coffee, rice, alcoholic, spicy), were not suitable for the type of product (snappy, creamy, oily) or simply hedonistic (harmonic). The term “chocolate” was discarded because it was statistically correlated with the cocoa aroma and was not legally appropriate for defining EVO bar (European Union, 2000). Another nine terms were discarded because of their low frequency of use after calculation of the geometric mean as indicated in ISO 11035:1994 (data not showed). The final list of 16 descriptors selected to be introduced in the product evaluation sheet is shown in Table 3.

**Table 2:** Complete vocabulary taken into consideration for the sensory description of Toscolata^®^ cocoa products and preparation of the evaluation Sheet

**Appearance**	**Taste and flavor**	**Texture**
Color^c^	Sour	Smoothness
Presence of crystals^c^	Bitter	Firmness
Bright	Sweet	Cohesive^c^
**Aroma**	Sapidity	Adhesive^c^
Cocoa	Nutty^b^	Melting^c^
Butter^b^	Coffee^b^	Friable^c^
Cinnamon^b^	Rice^b^	Gritty^c^
Vanilla^b^	Alcoholic^b^	Snappy^b^
Chocolate^b^	Persistence	Creamy^b^
Fruity	Tastiness^a^	Graininess
Smoky	Cinnamon^b^	Mealy^c^
Aromaticity^a^	Vanilla^b^	Oily^b^
Harmonic^b^	Chocolate^b^	
Vegetal^a^	**Mouthfeel**	
	Astringent	
	Spicy^b^	
	Rough^c^	

^a^Descriptors added by the assessors, ^b^eliminated after round table and the tasting, and ^c^after the calculation of the geometric mean.

**Table 3:** List of descriptors used in the Toscolata^®^ evaluation sheet

Appearance	Texture	Aroma	Flavor
Bright	Smoothness	Aromaticity	Sweet
	Graininess	Cocoa	Bitter
	Firmness	Fruity	Sour
		Vegetal	Astringent
		Smoky	Sapidity
			Tastiness
			Persistence

### 3.2 Panel test results

**Figure 1:**
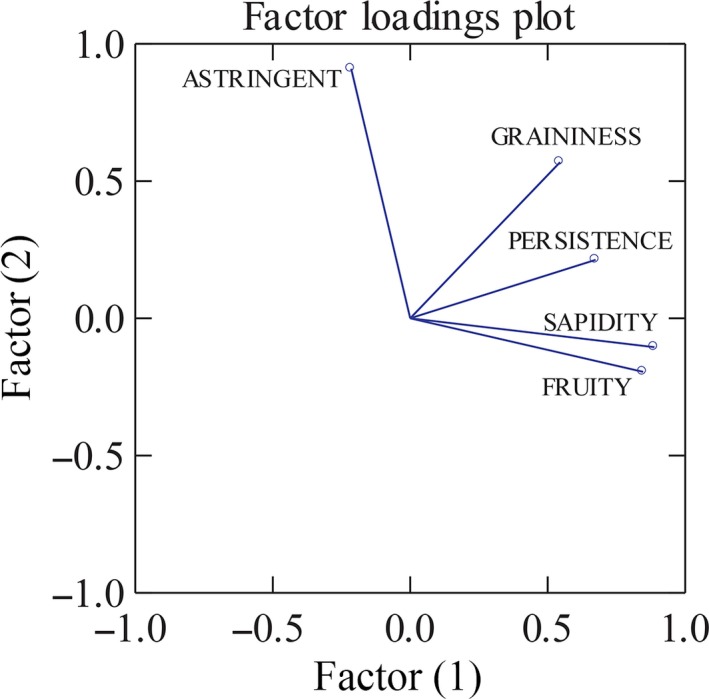
Factor loading plot of the most discriminant apple bars organoleptic attributes

**Figure 2:**
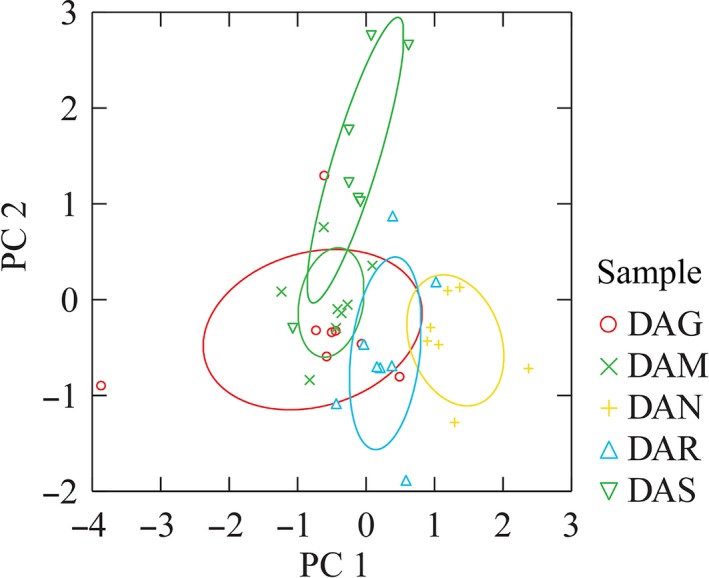
Plot of apple dried cocoa bar scores on the first two principal components. The ellipses represent the 95% confidence limits of each cocoa prototypes centroid

Data from the two separated sessions, statistically elaborated by PCA, underlined the most discriminant attributes of the sensory profile of cocoa bars only containing dried apples. The total variance explained by the first two principal components was equal to 71% and the five attributes with the highest loadings were “sapidity” and “fruity” on the first component and “graininess,” “astringency,” and “persistence” on the second component (Figure 1). A scatter plot of the scores of the five apple samples is shown in Figure 2. It possible to see from Figure 2 that some of the bars (DAG, DAM, and DAR) had similar scores on the first two components while DAS and DAN tended to differ from each other and from the rest. Since our interest was focused on Tuscan autochthonous apple varieties, the results of the panel, showing DAM and DAN highly separated by professional tasters was important because they probably were characterized by two different sensory profiles. In fact, the mean intensity of the sapidity were 3.9 ± 0.25 and 2.3 ± 0.20, respectively, in DAN and DAM. Persistence reached 3.8 ± 0.16 in DAN, while was lower (2.4 ± 0.15) in DAM. DAN presented the highest sapidity value (3.9) among the five apples–cocoa bars (DAG = 0.75 ± 0.16; DAS = 1.5 ± 0.21; DAM=1.35 ± 0.24; and DAR = 2.3 ± 0.18). DAM and DAN were the prototypes selected as representative of the novel bars containing dried apples to be used both in comparison to the other bars (CHF and EVO) and in the consumer test.

**Figure 3:**
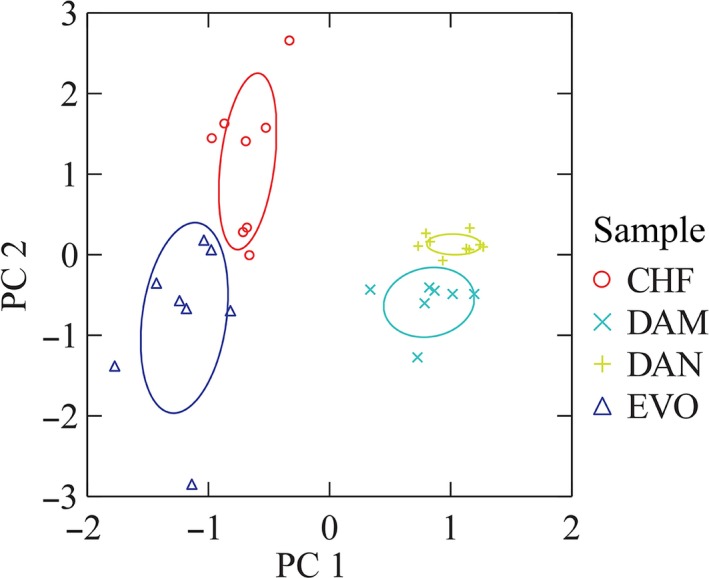
Plot of cocoa bar scores on the first two principal components. The ellipses represent the 95% confidence limits of each cocoa prototypes centroid

**Figure 4:**
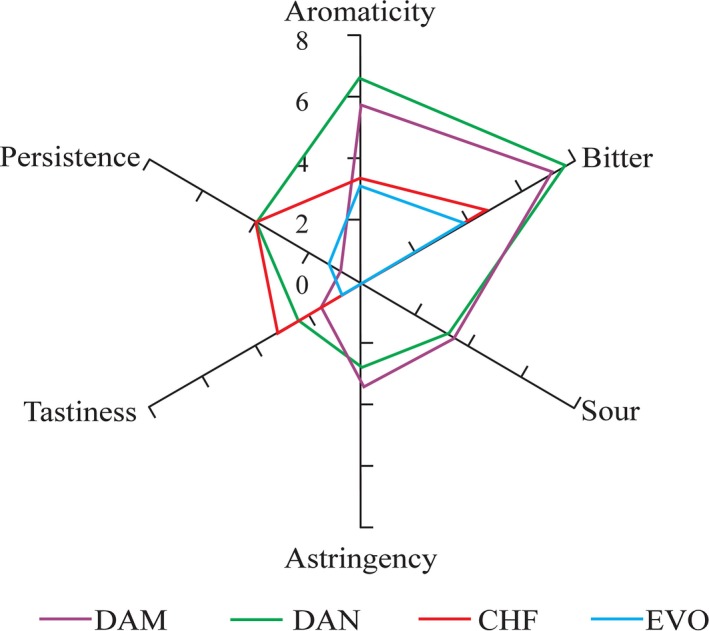
Graphic of quantitative descriptive attributes of the four cocoa prototypes

A new session with the professional panelists was conducted to profile the four final prototypes (DAM, DAN, EVO, and CHF) again using the sensorial sheet with all 16 attributes. The loading of each sensory property on the first two components as calculated by the Principal Component procedure is shown in Table 4. The “bitter” and “acidity” attributes contributed most in explaining the variation in the first component followed by “aromaticity” and “astringency” while in the second component “tastiness” and “persistence” were the most discriminant variables. The scatterplot of the scores of each sample on the first two PCs is reported in Figure 3. The four cocoa bars were well separated with DAM and DAN, both containing apple, being closed to each other and well distinguishable from EVO and CHF. The organoleptic profiles of the four cocoa bars as determined by professional panelists plotting the medians of these six most discriminant attributes are shown in Figure 4.

**Table 4:** Loadings of each attribute on the first two principal components calculated using the data produced by the panel test on the four cocoa bars

	Factor 1	Factor 2
Bright	0.817	0.248
Aromaticity	0.822	−0.034
Cocoa	0.777	0.033
Vegetal	0.073	0.124
Fruity	0.381	−0.007
Smoky	−0.373	0.441
Smoothness	0.596	−0.585
Graininess	0.011	0.335
Firmness	0.506	−0.141
Bitter	0.888	−0.093
Sweet	0.748	0.202
Sour	0.870	−0.220
Astringent	0.778	0.123
Sapidity	−0.254	0.309
Tastiness	0.270	0.804
Persistence	0.350	0.828

### 3.3 Consumer test results

The 182 consumers who participated in the test were well distributed within the classes of gender and age (Table 5), and regarding their preference, 51% liked unsweetened cocoa products. Only 15% of the consumers associated the purchasing of cocoa to health benefits.

**Table 5:** Background characteristics of the consumers participating at the consumer test (*N* = 182)

Background variable	%
Gender	
Male	47
Female	53
Age (year)s	
18–25	30
26–35	24
36–60	24
61+	22
Mood	
Quite	85
Not quite	15
Hunger	
Hungry	26
Neither/nor	19
Satiated	55
Cocoa preference	
Sweet	36
Neither/nor	14
Unsweetened	51
Purchasing of cocoa‐based products	
Everyday	12
Once a week	52
<2 times/month	36
Consumption of chocolate bars	
>6 bars	5
3–6 bars	17
1–2 bars	59
None	19
Choice of purchase	
Pleasure/food quality	73
Pleasure/food price	13
Healthy benefits/food quality	13
Healthy benefits/food price	2

**Figure 5:**
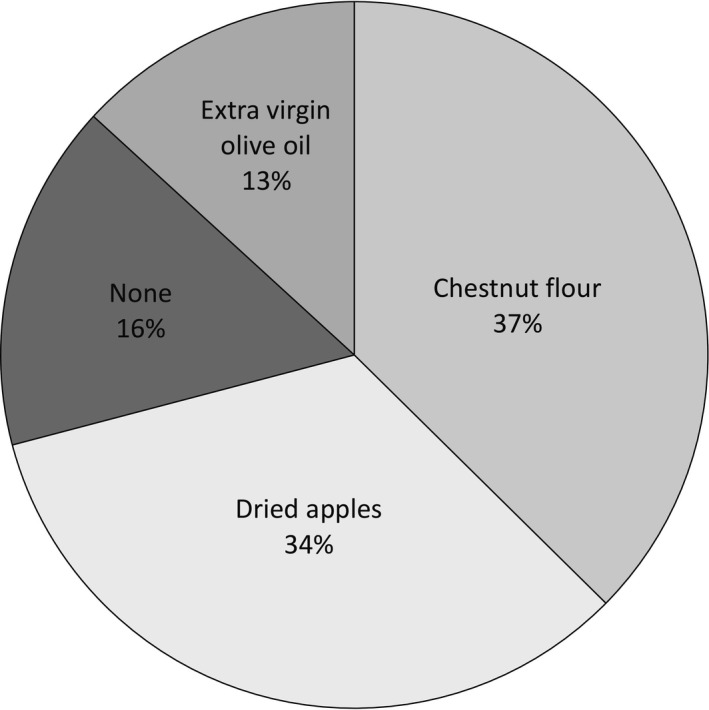
Distribution of the consumer preference among the cocoa bars with different ingredients

None of those interviewed disliked all the bars and only 16 (8.7% of the total) expressed to dislike (very or extremely) of one of the bars. The number of “dislike” opinion were, respectively, 8 for EVO, 5 for the apple bars, and 3 for CHF with no statistical differences among the number of answers. The preference of the consumers (Figure 5) was equally distributed between the bars containing chestnut flour (37%) and those containing dried apple (34%). This percentage takes into account both DAM and DAN as “apple” and the preference of the two was similar (43 vs. 57%). While 16% of the consumers did not express preference for any of the four (they liked all of them equally) only 13% of the total interviewed showed preference for the bar made with extra virgin olive oil.

We also tried to cluster the 182 consumers using the same employed in the PCA presented in Figure 1, and found that the three most discriminant variables for clustering were age, motivation, quantity of consumption. Only two clusters of consumers were obtained: one with both young and middle‐aged consumers that buy high quantities of chocolate for pleasure (1–2/3–6 bars/month) and a second including all the elders buying low quantities (1–2 bars/month) that were conscious of their health (Table 6).

**Table 6:** Distribution of cocoa bar preference within the consumers in relation to gender and age (*N *=* *182)

Cocoa bar	EVO					CHF					DAM+DAN					None				
	Count			%		Count			%		Count			%		Count			%	
	Total	M	F	M	F	Total	M	F	M	F	Total	M	F	M	F	Total	M	F	M	F
	24	12	12	50	50	68	38	30	56	44	61	23	38	38	62	29	11	18	38	62
Age																				
18–25	7	2	5	8	21	13	4	9	6	13	24	9	15	15	25	11	5	6	17	21
26–35	6	4	2	17	8	18	11	7	16	10	14	4	10	7	16	5	2	3	7	10
36–60	4	2	2	8	8	15	9	6	13	9	16	9	7	15	11	9	3	6	10	21
61+	7	4	3	17	13	22	14	8	21	12	7	1	6	2	10	4	1	3	3	10

Pearson Chi‐square preference content/sex *p *=* *.153.

Pearson Chi‐square age *p *=* *.418.

Pearson Chi‐square preference content/age *p *=* *.091.

Pearson Chi‐square preference content/age/sex *p *=* *.030 only for chestnut/male/61+.

With regard to the distribution of preferences among groups of consumers, the only statistical difference was found to be “male over 61 years old” who preferred the cocoa bar with chestnut flour (Pearson's chi‐square *p *=* *0.030).

We performed a calculation based on the consumers’ answers about the motivation for their preference, and the average score of the most highly used attribute for the four cocoa bars are reported in Table 7.

**Table 7:** Distribution of sensory attributes expressed to explain preference of the consumers toward each cocoa bar

Cocoa bar	EVO		CHF		DAM+DAN	
*N*	31		103		89	
		*p*		*p*		*p*
Cocoa aroma	10	ns	33	ns	29	ns
Bitter	5	ns	0	.05	15	.05
Sweet	6	ns	31	ns	9	ns
Texture	7	ns	14	ns	18	ns
Salty	1	ns	6	ns	5	ns
Other	2	ns	19	ns	13	ns

*p*, Pearson Chi‐square; ns, not significant.

## 4 DISCUSSION

As shown by Lanza et al. (2011), the selected specific descriptors for the products were able to profile the organoleptic features of the samples and to also discriminate among them. In contrast to “Modica” chocolate specialty, the appearance was not important in our case and only EVO was recognized as more “bright” than the other bars by the professional panelists. The four sensorial profiles produced by the experts for the cocoa bars differed in persistence, bitterness, aromaticity, acidity (sour), astringency, and tastiness. In particular, the sour attribute, mostly found in DAM and DAN, could be traced to the presence of dried apple. Also, in DAM and DAN, the bitter attribute was remarkable compared to the others. The bar with chestnut flour was characterized by its tastiness, while the natural presence of sugars gave sweetness or lowered the bitter sensation. As underlined by Thamke et al. (2009), consumers are limited in their use of attributes to describe dark chocolate. In our case, those interviewed, although with less accuracy in terms, were able to express a preference and only 11% of them did not selected a preferred bar. Our work showed that the selection of the preferred bar was mainly based on the presence or absence of the bitter attribute.

**Figure 6:**
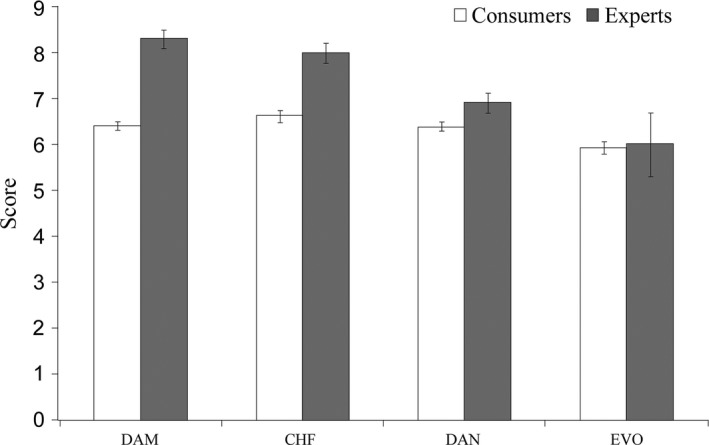
Bar plot comparing consumers and experts overall level of likeability expressed for each cocoa bar. The small bars represent the standard error of the mean

While similar research has been conducted for the assessment of apple quality by Gatti, Di Virgilio, Magli, and Predieri (2011), this paper is the first to compare the results of a panel with that of consumers for dark chocolate. Comparing the overall scores on likeability that experts and consumers expressed for each cocoa bar (Figure 6) is possible to notice only small difference in the results. The panelists mostly preferred the DAM followed by CHF and DAN chocolate bars, while the consumers preferred CHF followed by the bars containing apple (DAM and DAN) at the same level of appreciation. The appreciation of the bars containing extra virgin olive oil was the lowest for both professionals and consumers. Both of them found this bar “strange” without any other definition. Probably the panelists, most of whom did not recognize the ingredient, and which are mostly performing oil assessment felt an “alteration” in the fatty composition. They said that the cocoa bar was not defective but not pleasant. Those consumers who liked the EVO instead (*n* = 24) found this bar very “tasty” and pleasant in its smoothness, complexity, and persistency. With regard to EVO, we have to underline that the extra virgin olive oil is not considered by the European Commission directive among the vegetable oils/fats permitted in the production of chocolate, even though extra virgin olive oil is considered to be highly beneficial to health compared to palm oil which is instead included in the list of legal ingredients for chocolate. Recently, the Panel on Contaminants in the Food Chain (CONTAM) of the European Food Safety Agency (EFSA) evaluated the risks for human health related to the presence of 3‐ and 2‐monochloropropanediol (MCPD), and their fatty acid esters and glycidyl fatty acid esters in food. The EFSA reported that the esters of 3‐ and 2‐MCPD and glycidyl esters were highest in palm oil/fat, and that the level in some foods could cause health problem for young people. The EVO bar did not present any processing or conservation problem and has a peculiar organoleptic profile, different from other commercial products. Its action on human health is now under evaluation by our group. Since only 15% of the consumers declared that they buy cocoa for its effects on health, there is great potential for information strategies. The communication of scientific knowledge about these high‐quality products could be more greatly explored and exploited to increase market penetration and price.

## 5 CONCLUSION

The different ingredients used in the recipes directly influenced the acceptability of these novel bars to consumers. DAM, DAN, and CHF obtained higher acceptance ratings compared to EVO. This study demonstrated that novel food made with ingredients such as Tuscan autochthonous dried apples and PGI Monte Amiata chestnut flour had a high acceptance and preference by consumers.

## CONFLICT OF INTEREST

None declared.
